# Sustained Elevation of Systemic Oxidative Stress and Inflammation in Exacerbation and Remission of Asthma

**DOI:** 10.1155/2013/561831

**Published:** 2013-08-29

**Authors:** Judith C. W. Mak, Siu P. Ho, Alice S. S. Ho, Barbara K. W. Law, Amy H. K. Cheung, James C. M. Ho, Mary S. M. Ip, Moira M. W. Chan-Yeung

**Affiliations:** ^1^Division of Respiratory Medicine, Departments of Medicine, The University of Hong Kong, Hong Kong; ^2^Pharmacology & Pharmacy, The University of Hong Kong, Hong Kong; ^3^Research Centre for Heart, Brain, Hormone and Healthy Aging, The University of Hong Kong, Hong Kong; ^4^Room L8-40, Laboratory Block, LKS Faculty of Medicine Building, The University of Hong Kong, 21 Sassoon Road, Hong Kong; ^5^Alice Ho Miu Ling Nethersole Hospital, Tai Po, Hong Kong

## Abstract

Oxidative stress has been implicated in the pathogenesis of asthma. We aimed at investigating the biomarkers of oxidative stress, inflammation, and tissue damage in patients with asthma in acute exacerbation and remission. We recruited 18 asthmatics admitted to hospital with acute exacerbation and 18 healthy nonsmoking controls matched for age. We evaluated plasma levels of 8-isoprostane, C-reactive protein (CRP) and total matrix metalloproteinase- (MMP-) 9 by ELISA, and MMP-9 activity by zymographic analysis. Plasma levels of 8-isoprostane and CRP were significantly elevated in acute exacerbation and decreased in remission but remained significantly higher compared to healthy controls. The activities of pro-MMP-9 were also significantly higher in acute exacerbation and decreased in remission but remained significantly higher compared to healthy controls in parallel to plasma levels of total MMP-9. These data suggest that overproduction of MMP-9 along with highly elevated levels of oxidative stress and inflammation is implicated in asthma exacerbation and that measurements of these biomarkers can be a valid index in its management.

## 1. Introduction

Asthma is a chronic inflammatory airway disease that affects children and adults of all ages [1]. Chronic inflammation, which involves recruitment and activation of inflammatory cells, has been increasingly recognized as a mechanism leading to oxidative stress in asthma [2]. According to Wark and Gibson [3], the patterns of airway inflammation are different for different triggering factors, while the exaggerated response of asthmatic airways is still not completely well known. 8-Isoprostane is considered a marker of oxidative stress specific for lipid peroxidation, which is a stable metabolite of arachidonic acid, synthesized *in vivo*, and biologically active [4]. Previous studies found higher 8-isoprostane levels in the plasma, exhaled breath condensate (EBC), and induced sputum in asthmatic patients [5–7]. In the few studies carried out on patients in acute exacerbation of asthma, changes in oxidative stress have been reported, including increased plasma thiobarbituric acid reactive substances (TBARS), exhaled pentane levels, and exhaled breath 8-isoprostane in acute exacerbation versus stable asthma [8–10].

C-reactive protein (CRP), an acute-phase protein, is a highly sensitive systemic marker of inflammation, infection, and tissue damage [11]. Beside airway inflammation, several studies have indicated a positive relationship between asthma and increased CRP levels [12–17]. Recently, Fujita et al. found that serum CRP levels might be related to asthma exacerbations [18].

Airway remodeling, an excess of extracellular matrix (ECM) deposition in the airway wall, which leads to subepithelial collagen deposition, is a well-known feature of asthma. The matrix metalloproteinases (MMPs) belong to a large family of zinc- and calcium-dependent endopeptidases with distinct substrate specificities that degrade all types of ECM components. The MMPs play an important role in physiological and pathological processes, including ECM turnover, tissue degradation and repair, cell migration, and inflammation. Of the MMP family, MMP-9 is the major proteinase that induces airway remodeling in asthma [19]. MMP-9 is the most prominent MMPs found in bronchoalveolar lavage fluid, in sputum, and in serum [20–23]. It has been reported that eosinophils are a major cellular source of MMP-9 in bronchial tissue in asthmatic patients [24]. The levels of MMP-9 in sputum or in plasma have been related to the severity of asthma, with more severe asthmatics having higher levels than those with mild asthma [25, 26]. 

As acute exacerbation of asthma might contribute to airway remodeling by changing the levels of biomarkers for oxidative stress and inflammation, we therefore determined plasma levels of 8-isoprostane (biomarker of oxidative stress), CRP (biomarker of systemic inflammation), and total MMP-9 as well as MMP-9 activity in patients with asthma in acute exacerbation and in remission.

## 2. Materials and Methods

### 2.1. Study Subjects

Asthma is defined as those with a history of cough or wheeze and the presence of reversible airflow obstruction [27]. Eighteen patients with asthma admitted into two local hospitals with acute exacerbation were recruited by a research nurse within the first 24 hours of their admission. They all had received systemic corticosteroids within the first 24 hours of admission. A venous blood sample of 10 mL was collected before or shortly after the administration of systemic corticosteroids. A detailed questionnaire about their demographic characteristics was completed by the research nurse, and spirometry was carried out according to the American Thoracic Society recommendation [28] at the time. Another blood sample was taken, and spirometry was done again when the patients were in remission usually at 4 to 6 weeks after discharge from hospital. Eighteen patients were recruited from two local hospitals. In addition, 18 healthy nonsmoking subjects without respiratory symptoms were also recruited locally from the work place to serve as age-matched controls. All subjects had allergy skin prick test to one or more common allergens during the follow-up visit. A positive skin test reaction was defined as one with a wheal diameter equal or greater than 3 mm in comparison to the histamine positive and the saline negative control at 15 minutes after testing. 

Written informed consent was obtained from each participant of the study. This study was approved by the Ethics Committee of HKU/HA HKW IRB (UW 06-348).

### 2.2. Blood Samples

Fresh venous blood samples were collected into evacuated tubes containing lithium heparin and centrifuged at 1600 ×g for 10 minutes under 4°C. The plasma and buffy coat were separated.

### 2.3. Measurement of Plasma 8-Isoprostane, C-Reactive Protein (CRP), and Matrix Metalloprotease- (MMP-) 9

Levels of 8-isoprostane (Oxis Research, TX, USA), CRP (Diagnostic System Laboratories Inc., TX, USA), and total MMP-9 (R & D Systems) in plasma were measured using as commercially available enzyme-linked immunosorbent assay (ELISA) kits according to the manufacturer's instruction. All measurements were performed in duplicate and measured in adjacent wells to minimize assay variability.

### 2.4. Evaluation of Plasma Gelatinases (MMP-2 and MMP-9) Activities

MMP-2 and MMP-9 activities were analysed by zymography using 1 mg/mL gelatine as substrate. This method allows the determination of various types of MMPs on the basis of their molecular weight and allows the determination of their state of activation (latent and/or active forms).

Aliquots of plasma were subjected to electrophoresis on sodium dodecyl sulphate- (SDS-) polyacrylamide gel under nonreducing condition. After electrophoresis, gels were washed twice in 50 mM Tris-HCl (pH 7.6), 5 mM CaCl_2_, and 2.5% Triton X-100 for 1 h, rinsed abundantly with water, and then incubated in 50 mM Tris-HCl (pH 7.6), 5 mM CaCl_2_, 1% Triton X-100, and 0.02% NaN_3_ at 37°C for 18 h. The reaction was stopped by staining the gels with 0.1% Coomassie Brilliant Blue R250 and then destained in a solution of 10% acetic acid and 30% methanol. The molecular weights of gelatinolytic bands were estimated using recombinant protein molecular weight markers (10,000–225,000 Da) (Amersham Biosciences). The gelatinolytic activity was visualized as clear bands against a blue background, and the band intensity was quantified with a densitometric analyzer with GeneTools (Syngene, Frederick, MD, USA). 

### 2.5. Statistical Analysis

Variables were presented as mean ± standard deviation (SD) or median (interquartile range). Wilcoxon's matched-signed ranks test was used to conduct pairwise comparisons of the biomarkers between asthmatic groups at acute asthma attack and during remission. Mann-Whitney *U*-tests were used to compare the difference between asthmatic and healthy control groups. Correlations between plasma biomarker levels and other lung function parameters were determined by Spearman's rank correlation coefficient. SPSS for Windows version 14.0 (SPSS, Chicago, IL, USA) was used for statistical analyses where appropriate. A *P* value < 0.05 was considered statistically significant in all analyses.

## 3. Results

Eighteen atopic, nonsmoking patients with asthma, 5 males and 13 females, aged 18 to 61 years were included for this study. In addition, 18 healthy nonsmoking age-matched controls were recruited consisting of 6 males and 12 females, aged 18–65 years; of these, 9 were atopic and 9 nonatopic. The lung function parameters (FVC (% predicted) and FEV_1_ (% predicted)) but not FEV_1_/FVC ratio (%) significantly improved during remission compared with those during acute exacerbation and were significantly lower than healthy controls (Table 1).

**Figure 1:**
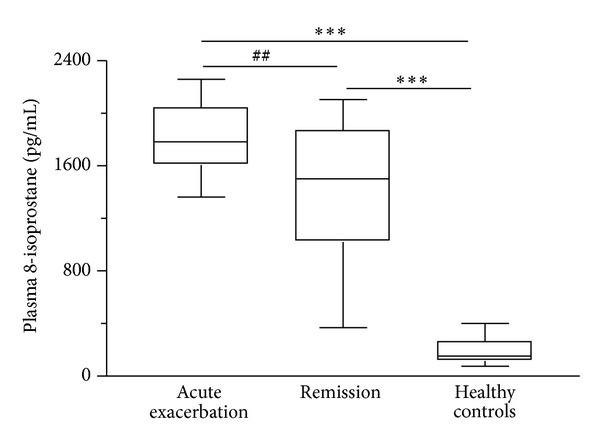
Box plots of plasma 8-isoprostane levels in asthmatic patients (*n* = 18) during acute exacerbation and remission and age-matched healthy controls (*n* = 18). The horizontal bars represent median values. ***P < 0.001 differences between acute exacerbation or remission and healthy controls; ^##^ *P* < 0.01 differences between acute exacerbation and remission by nonparametric Wilcoxon's rank sum test.

**Figure 2:**
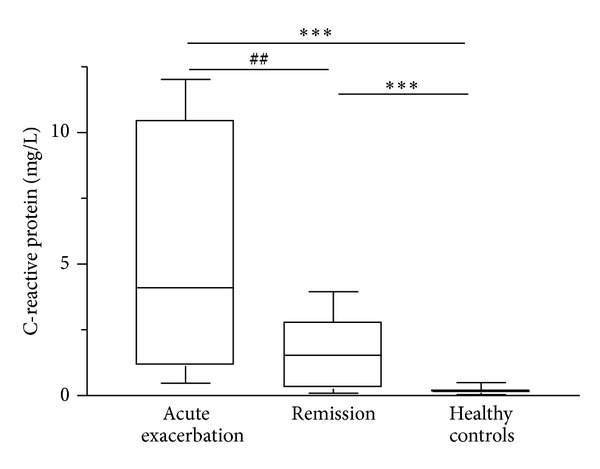
Box plots of plasma CRP levels in asthmatic patients (*n* = 18) during acute exacerbation and remission and age-matched healthy controls (*n* = 18). The horizontal bars represent median values. ***P < 0.001 differences between acute exacerbation or remission and healthy controls; ^##^ *P* < 0.01 differences between acute exacerbation and remission by nonparametric Wilcoxon's rank sum test.

**Figure 3:**
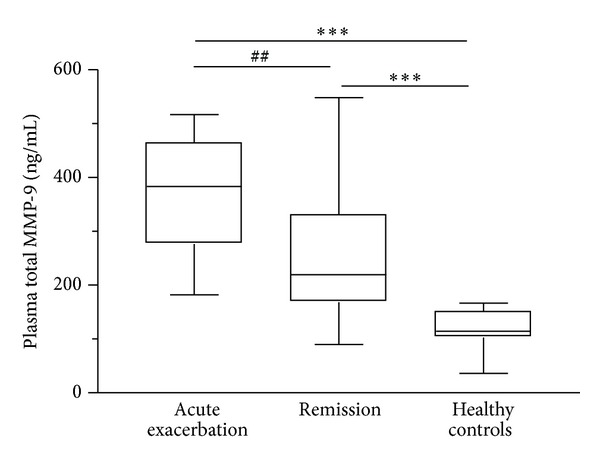
Box plots of plasma total (active and pro) MMP-9 levels in asthmatic patients (*n* = 18) during acute exacerbation and remission and age-matched healthy controls (*n* = 18). The horizontal bars represent median values. **P < 0.01 differences between acute exacerbation or remission and healthy controls; ^##^ *P* < 0.01 differences between acute exacerbation and remission by nonparametric Wilcoxon's rank sum test.

**Figure 4:**
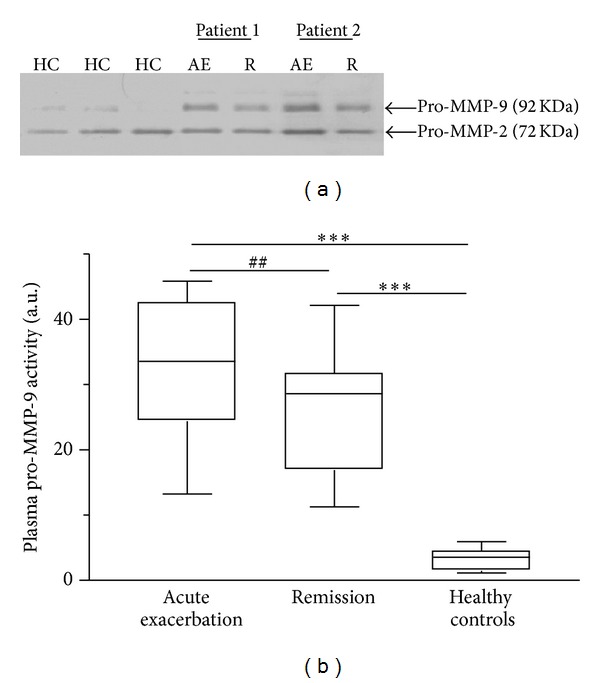
Analysis of plasma by gelatin zymography showing circulating gelatinolytic activities in healthy controls (HC) and their changes during an asthma acute exacerbation (AE) and subsequent remission (R). (a) A representative zymographic analysis of plasma samples from 3 healthy controls and 2 asthmatic patients suffering acute exacerbation and during remission. Two major bands are visible, corresponding to pro-MMP-2 (72 KDa) and pro-MMP-9 (92 KDa). Pro-MMP-9 activity is higher during an asthma acute exacerbation than during remission, whereas pro-MMP-2 activity remains constant. (b) Box plots of densitometric analyses of the zymographs as pro-MMP-9 activities. The horizontal bars represent median values. ***P < 0.001 differences between acute exacerbation or remission and healthy controls; ^##^ *P* < 0.01 differences between acute exacerbation and remission by nonparametric Wilcoxon's rank sum test.

Plasma levels of 8-isoprostane, CRP, and total MMP-9 were significantly higher in patients in acute exacerbation than those in remission or in healthy controls (*P* < 0.01; Figures 1, 2, and 3). Zymographic analysis showed the presence of the 72-KDa pro-MMP-2 and 92-KDa pro-MMP-9 bands (Figure 4(a)). We found a significant increase in activity of MMP-9 but not MMP-2 in the plasma of patients in acute exacerbation than those in remission or in healthy controls (*P* < 0.01; Figure 4(b)). 

We correlated plasma levels of each of the biomarkers with lung function parameters (FEV_1_ (% predicted), FVC (% predicted), and ratio of FEV_1_/FVC (%)) in acute exacerbation and in remission. There were a significant positive correlation between plasma 8-isoprostane levels and FEV_1_/FVC (*r* = 0.746, *P* < 0.001) and a significant negative correlation between plasma total MMP-9 levels and FEV_1_/FVC (*r* = −0.495, *P* < 0.05) in acute exacerbation but not in remission. No correlation was found between plasma CRP levels and any of the lung function parameters. A significant negative correlation also existed between the plasma levels of 8-isoprostane and total MMP-9 (*r* = −0.524, *P* < 0.05) in acute exacerbation but not in remission. 

## 4. Discussion

In this study we found that plasma levels of 8-isoprostane, CRP, total MMP-9, and 92-KDa MMP-9 activity were significantly increased in patients with asthma in acute exacerbation and decreased in remission but remained elevated compared with healthy controls. These findings suggest that there are a sustained systemic oxidative stress and inflammation and airway remodeling during recovery from acute exacerbation of asthma.

There is increasing evidence that asthma is a disease associated with increased oxidative stress [29]. In support, we found persistent elevated levels of 8-isoprostane during remission compared to healthy controls. One explanation could be its resistance to corticosteroids as demonstrated by Montuschi et al. [5] who reported that patients with severe asthma treated with oral prednisolone had higher 8-isoprostane levels in their exhaled breath condensates than patients with mild and moderate asthma.

CRP is one of the most characteristic surrogate markers of systemic inflammation. Our results confirmed the presence of low-grade systemic inflammation in asthma as shown previously [12–17]. In agreement with previous reports [18, 29], we found significantly higher plasma CRP levels at acute exacerbation and remained elevated during remission compared with healthy controls, reflecting chronic systemic inflammation in asthma. 

The findings of higher levels of MMP-9 levels in the proform (92 KDa) without changes in MMP-2 level in asthmatic patients at acute exacerbation than during remission in our study are also compatible with other investigators [26, 30] and suggest the involvement of MMP-9 in airway inflammation and remodeling. Inhaled steroids treatment has been found to decrease MMP-9 activity in some [20, 31, 32], but not in all, reports [25]. 

We found that the ratio of FEV_1_/FVC (%) was positively correlated with plasma 8-isoprostane levels while negatively correlating with total MMP-9, but there were no correlations between FEV_1_ or FVC (% predicted) and 8-isoprostane, CRP, total MMP-9, or its activity. Several previous studies have demonstrated an association between serum CRP and lung function parameters (FEV_1_ (% predicted), FVC (% predicted) or FEV_1_/FVC (%)) in asthma [14, 17, 18]. However, the positive correlation between the ratio of FEV_1_/FVC (%) and circulating 8-isoprostane is unexpected as weak association being observed between sputum 8-isoprostane and FEV_1_ (% predicted) in a previous study [7]. Further studies are needed to clarify whether the relationship between oxidative stress and airway obstruction exists in individual patients. The negative correlation between plasma levels of 8-isoprostane and total MMP-9 was consistent with previous report showing that 8-isoprostane reduces MMP-9 activity *in vitro* [33]. 

To the best of our knowledge, this is the first study to assess the plasma levels of 8-isoprostane, CRP, and total MMP-9 as well as MMP-9 activity in acute exacerbation and in remission of the same patients. It has been suggested that oxidative stress, inflammation, and airway remodeling would be best studied in cells and fluids obtained from the lungs such as induced sputum and BAL. We chose to study plasma for several reasons. Blood is an easily available source to study various biomarkers in asthmatic patients during acute exacerbation and remission. BAL can provide direct samples of airway cells, but the technique is invasive and can only be performed in stable asthmatic patients. Despite several confounding factors including aging and smoking, which might affect plasma levels of CRP, age-matched healthy controls for asthmatic patients were recruited, and smokers were excluded from this study. One limitation of this investigation is that the sample size being studied is rather small. We nevertheless believe that these data are meaningful and should prompt further large-scale investigations.

In conclusion, there is evidence of systemic oxidative stress and systemic inflammation in asthma exacerbation, which persists in remission. These findings suggest that overproduction of MMP-9 along with oxidative stress and inflammation is implicated in asthma exacerbation and that measurements of these biomarkers (for oxidative stress, inflammation, and remodeling) in blood can be a valid index in the management of asthma to assess the status of oxidative stress, inflammation, and remodeling.

## Figures and Tables

**Table 1:** Lung function parameters of asthmatics and healthy controls.

	Asthma		Control
	Exacerbation	Remission	
FVC (% predicted)	79.0 ± 24.3^∗,†^	99.0 ± 22.9	107.4 ± 14.7
FEV_1_ (% predicted)	62.6 ± 31.4^∗,†^	84.8 ± 26.1^‡^	108.4 ± 14.6
FEV_1_/FVC ratio (% predicted)	74.7 ± 19.1^†^	84.4 ± 11.3^‡^	101.2 ± 5.4

*P < 0.05 for comparison between acute exacerbation and remission in asthma.

^†^ *P* < 0.001 for comparison between asthmatics during acute exacerbation and healthy controls.

^‡^ *P* < 0.01 for comparison between asthmatics during remission and healthy controls.

## Abbreviations

CRP: C-reactive protein
ELISA: Enzyme-linked immunosorbent assay
FEV_1_: Forced expiratory volume in one second
FVC: Forced vital capacity.
